# Insights Into Co-Morbidity and Other Risk Factors Related to COVID-19 Within Ontario, Canada

**DOI:** 10.3389/frai.2021.684609

**Published:** 2021-09-13

**Authors:** Brett Snider, Bhumi Patel, Edward McBean

**Affiliations:** University of Guelph, Guelph, ON, Canada

**Keywords:** artificial intelligence, COVID-19, SHAP (shapley additive explanation), XGBoost (extreme gradient boosting), mortality, co-morbidity

## Abstract

The worldwide rapid spread of the severe acute respiratory syndrome coronavirus 2 has affected millions of individuals and caused unprecedented medical challenges by putting healthcare services under high pressure. Given the global increase in number of cases and mortalities due to the current COVID-19 pandemic, it is critical to identify predictive features that assist identification of individuals most at-risk of COVID-19 mortality and thus, enable planning for effective usage of medical resources. The impact of individual variables in an XGBoost artificial intelligence model, applied to a dataset containing 57,390 individual COVID-19 cases and 2,822 patient deaths in Ontario, is explored with the use of SHapley Additive exPlanations values. The most important variables were found to be: age, date of the positive test, sex, income, dementia plus many more that were considered. The utility of SHapley Additive exPlanations dependency graphs is used to provide greater interpretation of the black-box XGBoost mortality prediction model, allowing focus on the non-linear relationships to improve insights. A “Test-date Dependency” plot indicates mortality risk dropped substantially over time, as likely a result of the improved treatment being developed within the medical system. As well, the findings indicate that people of lower income and people from more ethnically diverse communities, face an increased mortality risk due to COVID-19 within Ontario. These findings will help guide clinical decision-making for patients with COVID-19.

## Introduction

With issues of the second wave of the COVID-19 pandemic ongoing in 2021 and the world in a continuing crisis, interest continues to escalate to improve the understanding of features resulting in virus caseload increases. In response, of particular interest are opportunities to improve modeling prediction capabilities which can provide more accurate information as it becomes available from the first and second waves of COVID-19. In this regard, until recently, data security and privacy issues have limited accessibility to alternate and detailed data sources, but opportunities are opening up and showing real potential. As an example, improved access to Ministry of Health for Ontario, enabled Snider et al. (2021) to develop powerful artificial intelligence (AI) models that are now able to predict mortality and recovery of COVID-19 patients with a high degree of accuracy; the models developed were based on data from Ontario Health Data Platform (February 22, 2020–October 20, 2020), utilizing extensive and detailed data for 57,390 individual COVID-19 cases.

AI models in general, and Snider et al. (2021) in particular, provide dimensions including the ability to uncover and understand the value of an array of “base” information, including co-morbidity data, that influence mortality rates including at the case-by-case level. Findings on the risks of mortality for individual patients have the potential to influence many important actions such as helping identify “most at-risk populations” thus providing insights on hospitalizations/medical strategies and opportunities to aid delivery of COVID-19 vaccination priority strategies in the future.

The findings and predictions made available from use of logistic regression and other AI models, have excellent potential, when caseload data are available. Specifically, the models of Snider et al. (2021) demonstrated excellent discrimination with all model’s area under the curve (AUC) exceeding 0.948, with the greatest being 0.956 for an XGBoost (Extreme Gradient Boosting) model. Hence, this paper advances the knowledge in mortality risk of COVID-19 patients in Ontario, Canada, by calculating and exploring SHapley Additive exPlanations (SHAP) values of parameters used for the XGBoost AI model developed by Snider et al. (2021). Most importantly, these models provide specifics on the causative/impactful inter-relationships, which allow extraction of additional information from datasets and exceed the information provided by logistics models since logistic models assume a specific type of relationship between input and output, whereas the machine learning models allow capture of a more flexible relationship. In order to see the exact form of the relationship, SHAP dependance plots were made and analyzed for 4 principal features driving the XGBoost mortality prediction model. Hence, these provide indications detailing the importance of the individual variables that can be used to characterize co-morbidities that can be important indicators as to whom may be most susceptible to mortality and more likely to be in need of intensive medical needs, arising from the COVID-19 virus. Also, the relationships identified using this approach between parameters such as co-morbidities and other risk factors associated with COVID-19, and the corresponding impact on the mortality prediction XGBoost model, provide information which can be of great value in designing effective non-pharmaceutical interventions (NPIs) and vaccination schedules.

## Review of Technical Literature

The impressive predictive capabilities of AI have resulted in AI models being adopted across a wide range of disciplines. Their excellent performance in some areas of investigation arises largely due to the ability of AI models to identify and to model complex patterns between input variables and the predicted output. However, the AI model’s complexity often makes it difficult to identify the relationships between the input variables and the output, resulting in most advanced AI models being classified as “black-boxes”.

These so-called black-box models can be very accurate in their predictions but leave the users wondering how individual factors contribute to the model’s final prediction. A number of dynamic and statistical models of COVID-19 outbreaks including SEIR models (which assign individuals to the susceptible (S), exposed (E), infected (I), and recovered (R) classes) have previously been used to study and analyze transmission (Hellewell et al., 2020; Tuite and Fisman, 2020; Kucharski at al., 2020). However, these epidemiological models require values for unknown parameters and rely on many assumptions (Hu at al., 2020). Interest in understanding how the individual factors contribute has resulted in a variety of interpretable machine learning techniques being developed in recent years to assist in the interpretation of the impact of specific input variables on the final prediction (Molnar, 2019). This information is critical in promoting the gaining of trust in the AI model, as well as providing insights into which variables are important, and identifying key relationships that influence the AI models’ final prediction.

AI models have played a major role during the COVID-19 pandemic, through COVID-19 case identification, predicting transmission scenarios, and identifying the mortality risks of specific COVID-19 patients (see e.g., Li et al., 2020a; Boulle et al., 2020; Li et al., 2020b; Dhamodharavadhani et al., 2020). A significant focus has been placed on ensuring these models are interpretable, to allow a better understanding of the factors contributing to the predictions of patients’ outcomes, and to help inform responses.

Some researchers have selected AI models that are interpretable by design, such as logistic regression and decision trees. Yan et al. (2020) used decision trees and blood samples to interpret and identify mortality prediction for COVID-19 patients using blood samples. Fisman et al. (2020) used logistic regression models to predict mortality risk of COVID-19 patients; their logistic regression model quantifies the weight of each input variable to the final prediction, making it straightforward to determine how the model is calculating the overall COVID-19 mortality risk. A similar study by Quiroz et al. (2021) developed a logistic regression model using clinical and imaging data from two hospitals in Hubei, China, for automated severity assessment of COVID-19 for individual patients, obtaining an AUC of 0.950 using a combination of clinical and imaging features. They interpreted the importance of features using SHAP values and found patients in severe conditions had co-morbidities which included cardiovascular disease, diabetes, hypertension and cancer which is similar to findings obtained from previous studies (see e.g., Petrilli et al., 2020; Richardson et al., 2020; Shi et al., 2020; Siordia, 2020). Thus, interpretable machine learning techniques help address the most significant limitation of machine learning i.e., the lack of transparency due to its’ black box nature, however, there are trade-offs between the accuracy of predictions and interpretability with such models (Du et al., 2020). Overall, interpretable AI algorithms such as logistic regression and decision trees allow for the user to identify the weights associated with the model’s input variables, but these approaches are often less accurate compared to black-box models (see e.g., Murdoch et al., 2019; Snider et al., 2021). For a critical discussion in a clinical context, see the work by Christodoulou et al. (2019).

Another technique is to apply model agnostic interpretation methods to black-box models to investigate the relationship between inputs and the model’s prediction. A leading agnostic method to interpret black box AI models is through the use of SHAP values (Molnar, 2019). Barda et al. (2020) explored their black-box mortality prediction model for Israel’s COVID-19 patients using SHAP values to estimate the contribution of individual features to the overall model predictions. The calculated SHAP values identified the importance of several demographic attributes that the model determined important in predicting COVID-19 mortality (for example, age and cardiovascular disease) but the model used by Barda et al. (2020) has limited individual-level data, making it difficult to explore key relationships between COVID-19 patients and mortality, such as income level and ethnicity.

## Materials and Methods

The following sections describe the datasets and models developed by Snider et al. (2021) to predict mortality risk of COVID-19 patients in Ontario, Canada. The SHAP value methodology and application used to explore the black-box prediction models are then outlined.

### Dataset Description

The Ontario Health Data Platform (OHDP) was used in this research to assemble extensive data regarding COVID-19 patients within Ontario. The OHDP dataset contains epidemiological and demographic information, recovery/mortality outcome information and co-morbidities of individuals residing in Ontario. The attributes which proved most useful by the AI models are listed in Table 1. Co-morbidities and age were collected from patient health records as of January 1, 2020; hence, diagnosis of additional medical conditions after this date were excluded. Of the 57,390 cases included in the dataset, 2,822 patients died of COVID-19 and the remaining 54,568 either recovered from COVID-19 or remained hospitalized as of January 1, 2021. Several input variables were derived using 2016 Canadian census data for the designated area of the individual patients. Canadian census location information is based on a size of approximately three blocks and hence is able to capture representation of ethnicity, income level and other social differences, and can therefore be considered robust. The census data includes: ethnic concentration (of residential area), commuter concentration, median income and household size (these values are unlikely to change significantly between date of census and start of pandemic). These values were converted into quintiles (division of the population into five equal-sized groups according to the distribution of input variables) with 1 being the lowest quintile, and 5 being the highest. Individuals with missing data were not included in these analyses. It is noted that long-term care (LTC) residents in Ontario did not include census-designated area information and therefore, data for the LTC residents were represented with a zero value.

**TABLE 1:** Characteristics of 57,390 Ontario residents with COVID-19.

Variable	Description	Range of values
Age	Age in years, as of Jan 1, 2020	0–105
Test date	Test date	Feb–Oct 2020
Sex	Indicator variable for sex	26,861 (M = 1, F = 0)
Hypertension	Chronic hypertension, as of Jan 1, 2020	15,778 (0,1)
LTC resident	LTC resident, as of Jan 1, 2020	5,179 (0,1)
Chronic_dementia	Chronic dementia diagnosed, as of Jan 1, 2020	4,746 (0,1)
Chronic_odd	Chronic diabetes diagnosed as of Jan 1, 2020	9,002 (0,1)
Ethnic concentration quint.	Calculated from Ontario marginalization index, based on census designation. Refers to visible minorities and/or recent immigrants	(0–5)^a^
Commuter concentration quint	% Of people that commute within census designated area - converted to quintiles	(0–5)^a^
Median income quint.	Median income within census-designated area - converted to quintiles	(0–5)^a^
Charl	Charlson co-morbidity index. Only 2,059 patients with charl above 0.	(0–10)
Household size quint.	Avg. Household size within census-designated area - converted to quintiles (5 being the highest, 0 = missing DA info).	(0–5)
CKD	Chronic kidney disease.	2,523 (0,1)
Cancer	Cancer index	2,995 (0–1)
Chronic_copd	Chronic obstructive pulmonary disease	4,030 (0–1)
Chronic_asthma	Asthma	9,100 (0–1)
Chronic_chf	Congestive heart failure	2,257 (0–1)
Stroke	If patient suffered a stroke previous to Jan 1, 2020	1,016 (0–1)
Cardiac ISCH	Cardiac ischemia	1,916 (0–1)
Rural	Indicator if a patient lives in a rural residence	1,746 (0–1)
Chronic_ra	Rheumatoid arthritis	567 (0–1)
Tia	Transient Ischemic Attack	722 (0–1)
immuno_comp	Immuno-compromised	237 (0–1)
Thala	History of Thalassemia	36 (0–1)
Cases recovered		54,568
Cases died		2,822

a (0 referring to missing information).

### Model Development

Snider et al. (2021) compared three black-box machine learning models which were 1) Artificial Neural Network (Venables and Ripley, 2002), 2) Random Forest (Wiens and Shenoy, 2018), 3) Extreme gradient boosting decision tree—XGBoost (Chen et al., 2021) and one interpretable machine learning model which was logistic regression (Venables and Ripley, 2002). These models were adopted because of their high accuracy in binary classification problems as well as their prevalence/adoption in previous literature. Prior to model calibration, the dataset was randomly split into two segments, namely an 80% training dataset and a 20% testing dataset where each model was calibrated using the training dataset and assessed for accuracy using the testing dataset. A grid search approach was used to adjust the hyper-parameters of the models using a 10-fold cross-validation technique repeated three times per model and optimized to produce the maximum area under the receiver operating characteristic curve (Area Under Curve, or AUC). The XGBoost model was determined to be the most accurate model, having an AUC of 0.956. Therefore, this paper explores the XGBoost model’s relationships between the input variables and the predicted mortality risk by calculating SHAP values for each attribute and patient included in the training dataset. Features such as the public health unit of individual cases from the same locality/region were excluded when training the model as such parameters could cause problems if a particular region has a higher number of patients compared to others.

### Shapley Additive Explanation Values

To explore the impact of each variable on the XGBoost’s mortality model prediction, SHAP values have been used. SHAP values determine the importance of a feature by comparing what a model predicts with and without the feature for each observation within the training data. Specifically, the SHAP values represent the final AI model’s prediction using the following equation:

$${\text{y}}_{i}{=\text{y}}_{\text{base}}+\text{f}\left({\text{x}}_{i1}\right)+\text{f}\left({\text{x}}_{i2}\right)+\text{…}+\text{f}\left(x_{iN}\right)$$

The *i* ^th^ sample (or patient) is defined as $x_{i}$, the N represents the final feature (or input parameter) for the *i* ^th^ sample (as defined by $x_{iN}$). The predicted value of the AI model is $y_{i}$ and the reference value, or mean value of the target sample variable, is defined as $y_{base}$. The function $f\left(x_{ij}\right)$ is the calculated SHAP value of $x_{ij}$. The SHAP values are calculated using SHAP for XGBoost R package (Liu and Just, 2020) and present the variable contribution on a log-odds scale (logarithm of the ratio of high mortality risk to low mortality risk).

## Results and Discussion

**FIGURE 1:**
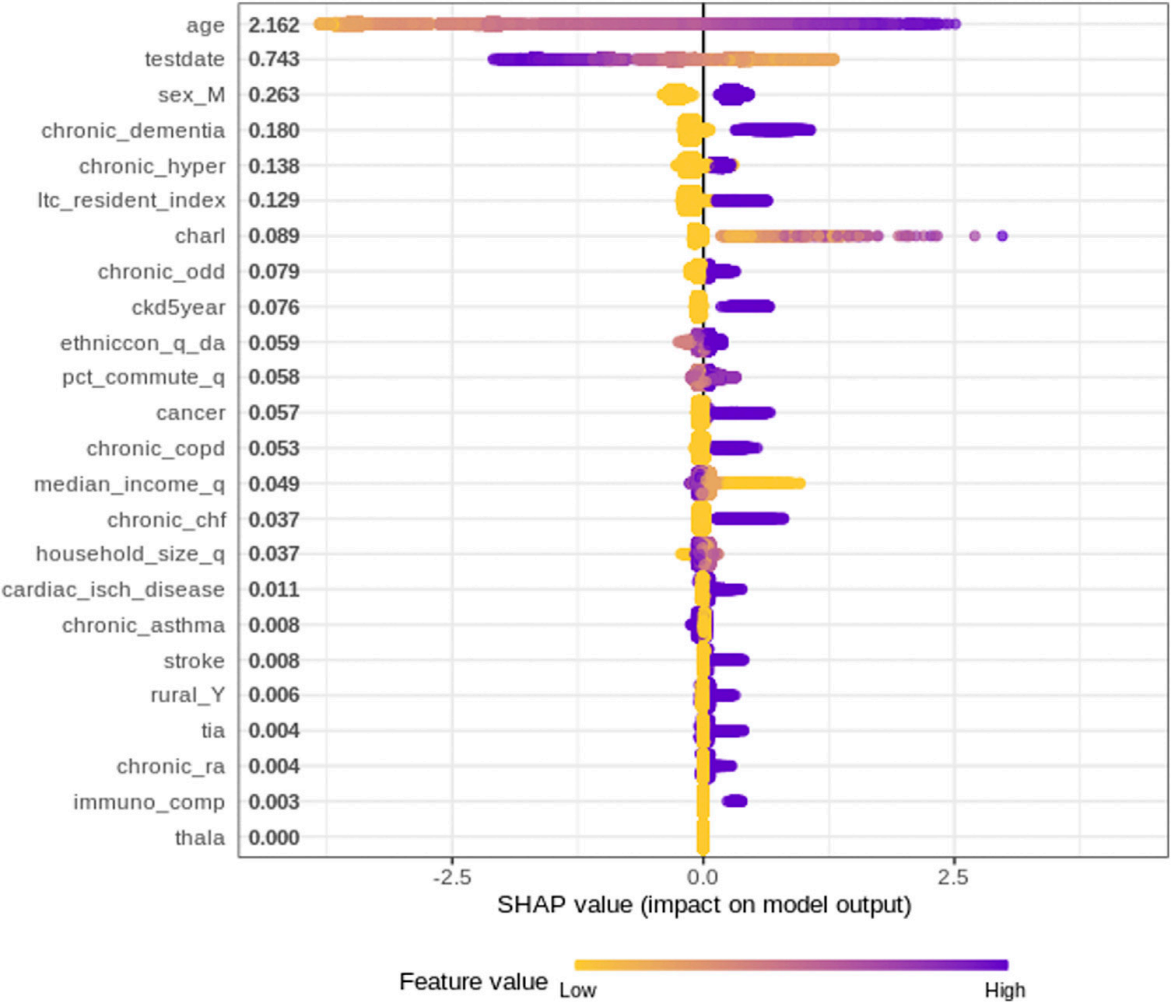
SHAP summary plot for XGBoost model.

Figure 1 plots the SHAP value for each individual patient within the training dataset for each input variable. The input variables, as listed on the y-axis, are ranked from most important (top) to least important (bottom) with their mean absolute SHAP value indicated next to the name in Figure 1. The X axis represents the SHAP value associated with each variable and patient within the training dataset (i.e., there is a plotted point for each case based on the influence that the variable has on the prediction of that case). The color indicates whether the individual patients’ input variable value was high (purple) or low (yellow). For example, in Figure 1 a “high” age has a high and “positive” impact on predicting mortality. The “high” comes from the purple color and the “positive” impact is shown along the X axis. Note, a range of SHAP values exists for each input variable value based on the SHAP values calculated for each observation, and how they independently contribute to the machine learning model’s predictions.

Overall, age is unquestionably the most important variable for the XGBoost model. As a patient’s age increases (approaches purple), the SHAP value impact increases, with a very high age being associated with an additional 2.5 increase in log-odds. The test-date when someone tested positive for COVID-19 also has a strong impact on overall mortality risk; as the positive test date increases (i.e., later on during the pandemic), the risk of mortality decreases.

The impact of the SHAP values are easily identified for binary variables, such as sex, hypertension, whether or not a patient was an LTC resident, and dementia. Being a Male (i.e., Sex = 1) has an additional 0.25 increase in log-odds, which indicates males have an increased risk of mortality. Similar increases are also identified with people having hypertension. An “LTC residence” designation is also associated with a significant increase in mortality, which is consistent with reported large numbers of outbreaks and deaths of individuals living in LTC homes. Chronic dementia is the co-morbidity associated with the largest increase in mortality.

### Age

**FIGURE 2:**
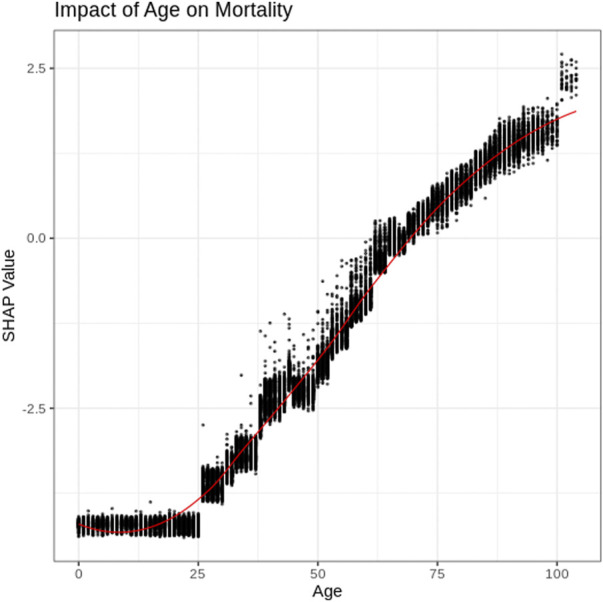
SHAP plot for Age.

The impact of a patient’s age on the AI model’s mortality risk prediction can be further explored using a SHAP dependency graph. Figure 2 depicts the SHAP values associated with patient ages within the training dataset. As further explanation of the results, a patient of <20 years of age is associated with a significant decrease in mortality risk; alternatively, as age increases, the risk of mortality increases. The non-linear shape of this figure, as well as the range of values for similar age highlight some of the advantages of the more complex AI models compared to less complex models such as logistic regression. Specifically, the XGBoost model examined here is able to identify complex patterns, as well as interaction effects that are often difficult for regression models (for example, logistic regression) to identify.

### Test Date

**FIGURE 3:**
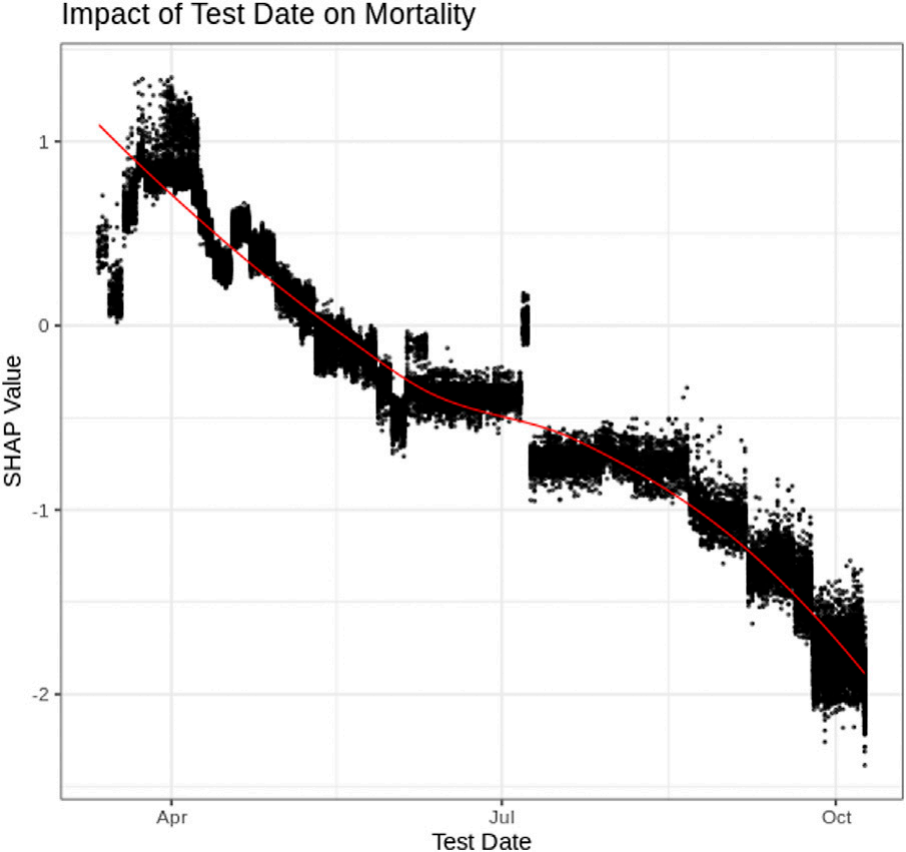
SHAP plot for Test Date.

Figure 3 depicts the SHAP values associated with the “day the patient tested positive” for COVID-19. Figure 3 indicates that residents of Ontario who tested positive for COVID-19 early on in the pandemic (e.g., April 2020) had an increased risk of mortality. From the data, mortality risk decreased for those individuals with later positive-test dates with a substantial decrease in mortality being associated with more recent months (e.g., September and October of 2020). Comparing positive test rates (% of tests performed that were positive) over the same time period identifies that “positivity rates increased during the period of substantial decrease in mortality” risk (October–December) (Public Health Ontario, 2021). This indicates that the decrease in mortality is unlikely a result of less severe cases being identified since positivity rates increased in October, while the associated risk decreased. Therefore, the decrease in mortality associated with later test date is considered more likely associated with improved treatment within the medical system (Robinson, 2021).

### Income

**FIGURE 4:**
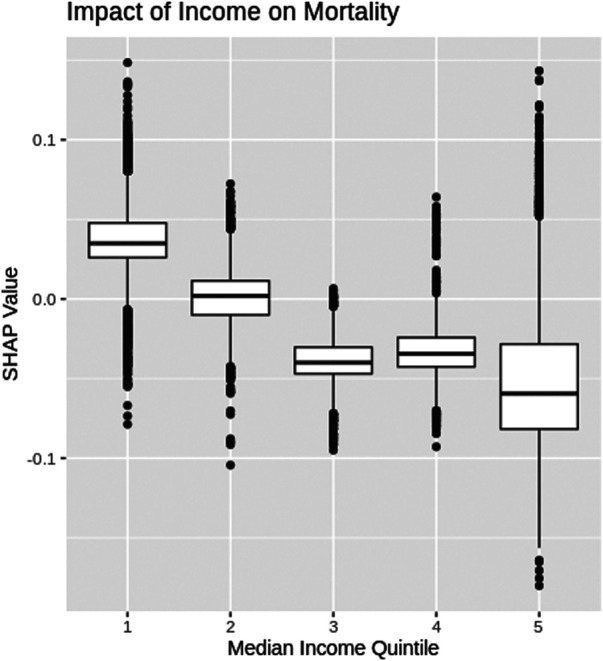
SHAP box-plot for median income.

The SHAP values for each median income quintile, based on census designated area, are depicted as box plots in Figure 4 (note income data were not available for LTC residents and therefore, LTC resident data were not included in Figure 4). COVID-19 patients who come from a census area with the lowest median income quintile have a higher risk of mortality. As the median income increases, Figure 4 shows the risk of COVID-19 mortality decreases.

### Ethnicity

**FIGURE 5:**
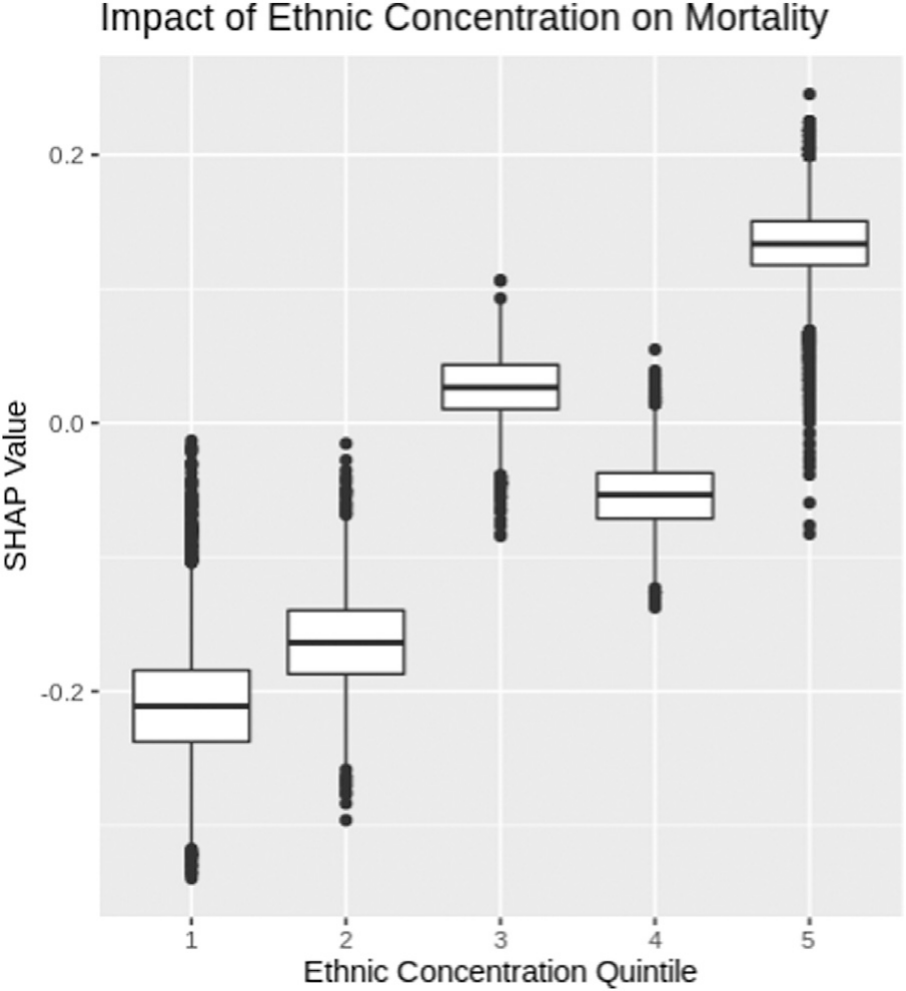
SHAP box-plot for ethnic concentration.

Ethnic concentrations were calculated based on 2016 census data for each designated area using the methodology outlined in the Ontario Marginalization index (Public Health Ontario, 2018). Specifically, ethnic concentration refers to the proportion of the population within a designated area who are recent immigrants or belong to a visible minority. The ethnic concentration was then segmented into quintiles and the SHAP values for each quintile are depicted using box plots in Figure 5. COVID-19 patients from census areas with high ethnic concentrations experience higher levels of mortality risk, while patients from neighborhoods with low ethnic concentrations experience lower levels of mortality risk. The ethnic and income factor results further highlight that COVID-19 has a greater impact among marginalized communities within Ontario, Canada.

## Conclusion

This paper explored an advanced AI mortality prediction model for COVID-19 patients within Ontario, Canada. Specifically, SHAP values were calculated and examined in order to uncover the relationships identified by the XGBoost model used by Snider et al. (2021). Several key findings are identified through this research. First, by examining the average SHAP value for each variable, key attributes related to mortality risk are identified (Figure 1). Age and test date are determined to be the leading factors that influence the mortality risk of COVID-19 patients in Ontario but also identified as important were sex, dementia, ethnicity, etc. at lesser degrees of importance.

SHAP dependency graphs are shown to provide very useful interpretation of the black-box XGBoost mortality prediction model. This paper explores four key attributes using SHAP dependency graphs: patients’ age, test-date, income and ethnic concentration. The Age SHAP dependency plot highlights the non-linear relationship between the patients age and risk of COVID-19 mortality, highlighting the significant increase in mortality risk associated with older patients with COVID-19 in Ontario. The Test-date dependency plot indicates mortality risk has dropped substantially within Ontario since the start of the pandemic. The SHAP values for income and ethnic quintiles suggests people of lower income and higher ethnic concentrations face an increased mortality risk due to COVID-19 within Ontario. Further exploration into these trends will be important as vaccinations become more widespread around the world, and with variants of concern becoming more common.

Overall, AI models have and will continue to play a major role in understanding and combating the COVID-19 pandemic. However, to build trust in these models and to gain further insight, a strong emphasis must be placed on ensuring the results from these models are interpretable. SHAP values are shown to be a useful tool to “open up” some of the more complex black box AI models and uncover the key patterns being modeled. The findings gathered from the model exploration performed in this paper further adds to the literature regarding mortality risks associated with COVID-19 patients and will help guide strategic interventions and vaccination schedules.

## Data Availability

These datasets were linked using unique encoded identifiers and analyzed at ICES. The use of the data in this project is authorized under section 45 of Ontario's Personal Health Information Protection Act (PHIPA) and does not require review by a Research Ethics Board. Access to datasets: The dataset from this study is held securely in coded form at ICES. While legal data sharing agreements between ICES and data providers (e.g., healthcare organizations and government) prohibit ICES from making the dataset publicly available, access may be granted to those who meet pre-specified criteria for confidential access, available at www.ices.on.ca/DAS (email: das@ices.on.ca). The full dataset creation plan and underlying analytic code are available from the authors upon request, understanding that the computer programs may rely upon coding templates or macros that are unique to ICES and are therefore either inaccessible or may require modification.

## Author Disclaimer

The analyses, opinions, results, and conclusions reported in this paper are those of the authors and are independent from the funding sources. No endorsement by ICES, the OHDP, its partners, or the Province of Ontario is intended or should be inferred. Parts of this material are based on data and/or information compiled and provided by CIHI. However, the analyses, conclusions, opinions, and statements expressed in the material are those of the author(s), and not necessarily those of CIHI. Parts of this material are based on data and information provided by Cancer Care Ontario (CCO). The opinions, results, view, and conclusions reported in this paper are those of the authors and do not necessarily reflect those of CCO. No endorsement by CCO is intended or should be inferred.
