# Corrigendum: Insights Into Co-Morbidity and Other Risk Factors Related to COVID-19 Within Ontario, Canada

**DOI:** 10.3389/frai.2021.759022

**Published:** 2021-09-13

**Authors:** Brett Snider, Bhumi Patel, Edward McBean

**Affiliations:** School of Engineering, University of Guelph, Guelph, ON, Canada

**Keywords:** artificial intelligence, COVID-19, SHAP (shapley additive explanation), XGBoost (extreme gradient boosting), mortality, co-morbidity

In the original article, there was an error. The dataset we analyzed requires very specific language regarding acknowledgements. We have therefore adjusted the corresponding text in the acknowledgments, disclaimer and data availability sections

A correction has been made to the **Acknowledgments** section:

“This study was supported by ICES, which is funded by an annual grant from the Ontario Ministry of Health (MOH) and the Ministry of Long-Term Care (MLTC). This study was supported by the Ontario Health Data Platform (OHDP), a Province of Ontario initiative to support Ontario’s ongoing response to COVID-19 and its related impacts. We thank IQVIA Solutions Canada Inc. for use of their Drug Information Database. Ontario Community Health Profiles Partnership (OCHPP) created Ontario Marginalization Index (ON-Marg) which is a source for this paper as ON-Marg is used to understand inequalities in health and other social problems related to health among either population groups or geographic areas across Ontario. Postal Code Conversion File and census data were adapted from Statistics Canada. This does not constitute an endorsement by Statistics Canada of this product.”

A correction has been made to the **Author’s Disclaimer** section:

“The analyses, opinions, results, and conclusions reported in this paper are those of the authors and are independent from the funding sources. No endorsement by ICES, the OHDP, its partners, or the Province of Ontario is intended or should be inferred. Parts of this material are based on data and/or information compiled and provided by CIHI. However, the analyses, conclusions, opinions, and statements expressed in the material are those of the author(s), and not necessarily those of CIHI.

Parts of this material are based on data and information provided by Cancer Care Ontario (CCO). The opinions, results, view, and conclusions reported in this paper are those of the authors and do not necessarily reflect those of CCO. No endorsement by CCO is intended or should be inferred.”

A correction has been made to the **Data Availability Statement** section:

“These datasets were linked using unique encoded identifiers and analyzed at ICES. The use of the data in this project is authorized under Section 45 of Ontario’s Personal Health Information Protection Act (PHIPA) and does not require review by a Research Ethics Board.

Access to datasets: The dataset from this study is held securely in coded form at ICES. While legal data sharing agreements between ICES and data providers (e.g., healthcare organizations and government) prohibit ICES from making the dataset publicly available, access may be granted to those who meet pre-specified criteria for confidential access, available at www.ices.on.ca/DAS (email: das@ices.on.ca). The full dataset creation plan and underlying analytic code are available from the authors upon request, understanding that the computer programs may rely upon coding templates or macros that are unique to ICES and are therefore either inaccessible or may require modification.”

The authors apologize for this error and state that this does not change the scientific conclusions of the article in any way. The original article has been updated.

